# British Dental Association

**Published:** 1905-01

**Authors:** 


### BRITISH DENTAL ASSOCIATION.

We are in receipt of a letter from the Secretary of the British Dental Association, W. H. Dolamore, of which the following is the substance:

"I have the honor to inform you that at a meeting of the Representative Board of this Association, held on October 29, it was resolved: That an invitation be extended to the National Dental Association of the United States, the Dental Association of the Dominion of Canada and other British Colonies, to attend the annual meeting of the British Dental Association, to be held at Southport, on Saturday, May 20, Monday, 22, and Tuesday, 23, 1905."

Whether it will be possible for the Executive Committee of the National Dental Association to accept this invitation remains to be seen. It is to be hoped it will devise some plan to secure the sense of that body, for it seems to the writer very important that this should be accepted at this time. Every effort should be made to draw the English-speaking race closer together, and this is one of the means to accomplish this professionally.

